# Ionomeric Nanofibers: A Versatile Platform for Advanced Functional Materials

**DOI:** 10.3390/polym16243564

**Published:** 2024-12-20

**Authors:** Mrinal Poddar, Yen-Hsiang Chang, Fang-Chyou Chiu

**Affiliations:** 1 Department of Chemical and Materials Engineering, Chang Gung University, Taoyuan 333, Taiwan; d1123007@cgu.edu.tw; 2 Department of General Dentistry, Chang Gung Memorial Hospital, Taoyuan 333, Taiwan; chy4d25@cgmh.org.tw; 3 Department of Chemical Engineering, Ming Chi University of Technology, New Taipei City 243, Taiwan

**Keywords:** nanofibers, ionomers, ionomeric nanofibers, one-dimensional nanomaterial, ionic functional group, electrospinning, ion exchange membrane, proton exchange membrane, innovative fabrication technique

## Abstract

The one-dimensional nanomaterials known as nanofibers have remarkable qualities, such as large surface areas, adjustable porosity, and superior mechanical strength. Ionomers, types of polymers, have ionic functional groups that give them special properties, including high mechanical strength, water absorption capacity, and ionic conductivity. Integrating ionomers and nanofibers with diverse materials and advanced methodologies has been shown to improve the mechanical strength, processing capacity, and multifunctional attributes of ionomeric nanofibers. One-dimensional ionomeric nanomaterials offer a versatile platform for developing functional materials with ionic functionalities. This mini review critically examines recent progress in the development of ionomeric nanofibers, highlighting innovative fabrication techniques and their expanding applications across energy storage, environmental remediation, healthcare, advanced textiles, and electronics.

## 1. Introduction

### 1.1. Nanofibers

Nanofibers, one-dimensional nanomaterials with diameters between 20 and 200 nanometers, have surfaced as a potential class of materials offering distinct properties for use in various fields [1,2]. These include their elongated shape, wound-healing capabilities, potential for use in tissue engineering, and ability to form complex 3D structures [3,4]. Nanofibers can be prepared from various materials, including carbon-based composites, metals, metal oxides, and natural/synthetic polymers [1,5]. The classification of nanofibers based on rigidity, composition, nature, and structure allows for tailored properties and different applications [6].

**Figure 1:**
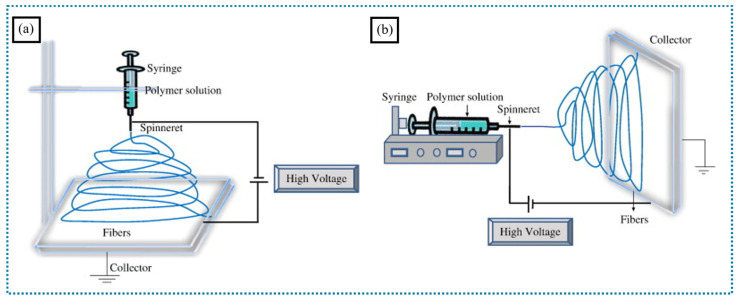
Schematic of the electrospinning setups: (**a**) vertical and (**b**) horizontal setups (adapted with permission from [7], Copyright © 2010 Elsevier Inc.).

Among the various processes for producing nanofibers, electrospinning has been studied the most because it allows the creation of nanofibers with higher surface-area-to-volume (SA/V) ratios and a significant number of inter/intra pores (Figure 1a,b) [7]. The ongoing research and development regarding electrospinning have led to increased debate regarding the use of laboratory-scale equipment versus the introduction of innovative production techniques to address low productivity [8]. Ongoing efforts focus on improving nanofiber alignment during deposition, creating more complex architectures for enhanced cellular adhesion, increasing mat thickness, incorporating bacteria and multistage release matrices, and overcoming the limitations of electrospinning through multilayer nanofiber production [3]. Industrial productivity has been enhanced through the development of more advanced nanofiber synthesis techniques, including modifications to traditional electrospinning methods like needleless and edge electrospinning [9,10,11,12].

Selecting the suitable polymer for nanofiber fabrication is critical for tailoring the properties of nanofibers to specific applications. Polymers should ideally be mechanically strong, biodegradable, safe, and mildly hydrophilic in nature. The origins of polymers used in nanofiber synthesis can vary from natural to synthetic, each offering its own advantages and disadvantages. Polymer blend nanofibers, composed of various polymer combinations, have shown promising results in regenerative medicine, serving as tissue-engineering scaffolds, wound dressings, and vascular grafts. They also find applications in targeted delivery and release of biomolecules. The high aspect ratios of these nanofibers lead to mats with large pore volumes and diverse pore sizes, offering significant mechanical strength while remaining lightweight [13].

Their unique properties, such as high SA/V ratios, biocompatibility, and a porous structure, make them ideal for various fields. In the biological realm, nanofibers can improve cell adhesion, growth, and differentiation, mimicking the extracellular matrix [14,15]. They can also be used for drug delivery, encapsulating and releasing drugs in a controlled manner. In filtration and separation, nanofiber-based membranes can remove contaminants while also allowing smaller particles or liquids to pass through [16,17,18]. Affinity membranes, designed to selectively bind to specific molecules, are particularly useful for purification and separation [19,20]. Beyond these applications, nanofibers are also utilized in tissue engineering as scaffolds for tissue regeneration, wound dressings due to their ability to absorb exudates and promote cell migration, and protective clothing to allow enhanced protection against harmful substances [21,22]. Modified-structure and composite nanofibers have been applied in electrochemical energy storage and conversion and flexible electronics [23,24]. The versatility of nanofibers has made them a subject of intense research and development, leading to numerous innovative applications across various industries. The main advantages and disadvantages of nanofibers have been listed in Table 1.

### 1.2. Ionomers

Ionomers are polymers containing a low concentration of ionic groups along their chains [25,26]. Unlike some ion-exchange resins, which are insoluble due to their crosslinked structures, ionomers achieve insolubility through the formation of crystalline domains [27,28]. The presence of ionic groups in their structures significantly enhances ionomers’ mechanical properties, shifts their glass transition temperatures, and modifies their diffusion properties. The main advantages and disadvantages of ionomers have been listed in Table 2.

Ionomers can be classified into two main groups based on their charge carriers: cation-conducting and anion-conducting polymers. Cation-conducting polymers possess negatively charged groups (like sulfonic or carboxylic acid groups) that bind to positively charged ions, such as protons (H^+^) or potassium ions (K^+^). These ions can then move through the polymer matrix, facilitating electrical conductivity. Anion-conducting polymers, conversely, contain positively charged groups (like quaternary ammonium groups) that attract negatively charged ions, such as hydroxide (OH^−^) or chloride (Cl^−^) ions. These ions can migrate through the polymer, enabling ionic conduction [29].

Ionic groups in ionomers tend to aggregate due to their lower dielectric constants compared to those of other organic polymers and attractive electrostatic forces. This occurs because of the existence of ionic groups that are oppositely charged, which attract one another electrostatically and lead them to form aggregates or clusters. The covalent connection between the polymer chain and the ionic groups, however, restricts this aggregation. Customizing ionomers for particular purposes requires an understanding of the limit of association, aggregate structure, and the variables influencing these processes [26].

There are numerous types of ionomers, each offering unique properties and applications. Hydrocarbon-based ionomers like sulfonated poly(arylene ether sulfone) (SPES) and sulfonated poly(ether ether ketone) (SPEEK) are commonly used due to their good thermal and chemical stability [30,31]. However, perfluorosulfonic acid (PFSA) ionomers, such as Nafion^®^, Flemion^®^, and Aciplex^®^-F, are particularly noteworthy because of their outstanding properties and broad range of applications [32,33,34]. These materials possess excellent chemical stability, high proton conductivity, and selectivity, making them ideal components in various electrochemical devices, including polymer electrolyte fuel cells (PEFCs), chlor-alkali cells, and water electrolyzers [33,35,36].

PFSA ionomers feature a hydrophobic polytetrafluoroethylene backbone with hydrophilic sulfonic acid side chains attached through perfluoroether linkages. Despite their lower ion exchange capacity (IEC) than hydrocarbon ionomers, PFSA ionomers exhibit micro-phase separation due to the chemical dissimilarity between the backbone and side chains [37]. This separation creates a network of hydrophilic ion-conducting channels within a hydrophobic matrix. When hydrated, these channels enable high proton conductivity. Additionally, the perfluorinated structure of PFSA ionomers imparts exceptional oxidative and chemical stability, making them ideal for demanding electrochemical applications.

While PFSA ionomers have dominated the fuel cell market, hydrocarbon ionomers offer several advantages. These include synthetic versatility, tunable properties, low gas permeability, a high glass transition temperature, and lower cost [29]. Sulfonated aromatic hydrocarbon polymers, such as poly(ether ether ketone), poly(ether sulfone), polyimides, and polybenzimidazoles, are promising alternatives to PFSA membranes due to their excellent mechanical properties, the ease with which they can be processed, and high chemical and thermal stability [38,39,40,41]. These ionomers can be synthesized via either post-polymerization sulfonation or direct synthesis using sulfonated monomers. The degree of sulfonation, which influences the ion exchange capacity, is crucial for achieving high proton conductivity [42].

### 1.3. Ionomers and Nanofibers: A Synergistic Partnership

When coupled, ionomers and nanofibers can provide a potent synergy that overcomes the drawbacks of each material alone. Because of their unique qualities and potential for a wide range of applications, electrospun ion-conducting nanofibers have attracted a lot of attention. A few of the possible advantages are listed below.

- Improved mechanical qualities: Nanofibers can reinforce an ionomer matrix, increasing its modulus, toughness, and tensile strength. This can aid in overcoming the restricted mechanical characteristics and brittleness that ionomers may have.
- Improved fiber stability: Ionomers have the capacity to create ionic connections between electrospun nanofibers, which increases their stability and keeps them from merging or clumping together.
- Better processability: By increasing ionomers’ dispersion and decreasing their viscosity, nanofibers can make it easier to process ionomers.
- Tailored functional properties: Nanofibers can be functionalized with various chemical groups to exhibit properties specific to the ionomer composite. This allows for the preparation of materials with tailored properties for diverse applications. Ionomers can be chosen with specific ionic functional groups to impart desired properties to the nanofibers, such as ion-exchange capabilities, pH-responsiveness, or improved biocompatibility.
- The following sections will delve deeper into the different properties and applications of ionomeric nanofibers.

## 2. Ionomeric Nanofibers

Ionomeric nanofibers are special types of nanofibers that contain both electrically neutral and ionized units within their nanofiber structures. These ionized groups, often carboxylic acid groups, give ionomeric nanofibers distinctive properties, such as ionic conductivity and the capability to form ionic-rich domains. Accordingly, the morphology of ionomeric nanofibers, characterized by their shape, size, and structure, becomes important in determining their performance and functionality. Several studies have investigated the factors influencing ionomeric nanofiber morphology, including polymer type, solvent composition, and processing methods.

**Figure 2:**
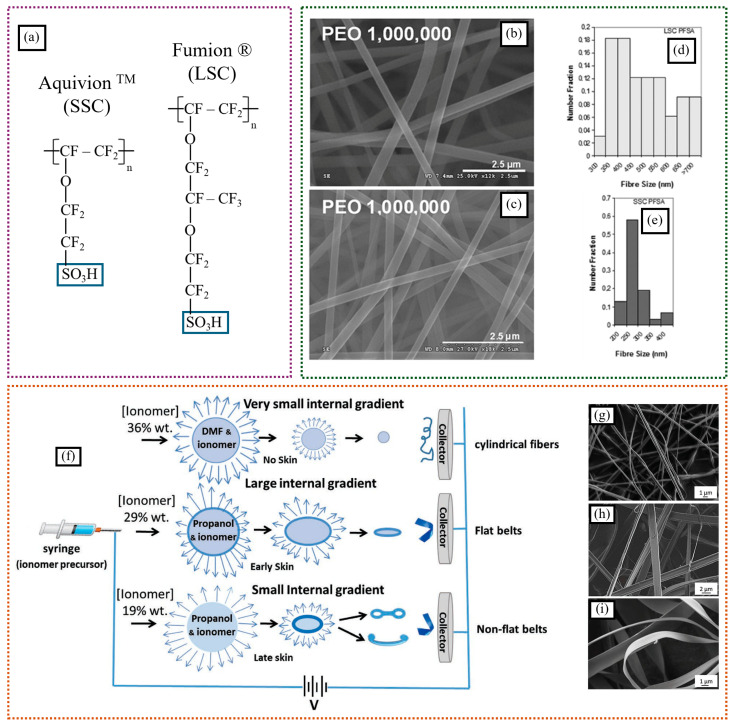
(**a**) Structures of LSC and SSC–PFSA ionomers. SEM images of (**b**) Fumion^®^ and (**c**) Aquivion^TM^ nanofiber mats made from 20% dispersion in DMAc with 1.5% and 1% (*m*/*v*) PEO (M.W.—1,000,000), respectively. Fiber size distribution for the same fibers: (**d**) Fumion^®^ and (**e**) Aquivion^TM^ (adapted with permission from reference [43], Copyright © 2012 Wiley Periodicals, Inc.) (**f**) Schematic for the preparation of ionomer fibers with different solvent and ionomer contents. HR-SEM micrographs of anion-conducting nanofibers: (**g**) cylindrical (36 weight% FAA-3, in DMF), (**h**) flat ribbon-like (19 weight% FAA-3, in propanol), and (**i**) flat ribbon-like (29 weight% FAA-3, in propanol) (adapted with permission from reference [44], © 2019 WILEY-VCH Verlag GmbH & Co. KGaA, Weinheim).

One key factor affecting nanofiber morphology is the side chain length of the ionomer. Subianto et al. studied the effect of the lengths of the side chains of perfluorosulfonic acid ionomers on the structural and dimensional properties of electrospun nanofiber composites and ionic conductivity [43]. They tested long- (Fumion^®^) and short-side-chain (Aquivion^TM^) commercial ionomeric polymers (Figure 2a) along with a sacrificial base polymer (polyethylene oxide, PEO). They examined the changes in fiber morphology in terms of variable ratios of ionomer to base polymer, different molecular weights of the base polymer, and different solvent compositions (DMAc (*N*,*N*-dimethylacetamide), water, isopropyl alcohol (IPA), and methanol). Compared to Fumion^®^, Aquivion^TM^, at an identical PFSA concentration of 20%, formed thinner fibers with a narrower size distribution (Figure 2b–e). This can be attributed to two factors: the denser packing of SSCs in Aquivion^TM^ during electrospinning, and the possibility of electrospinning Aquivion^TM^ at higher PFSA:PEO ratios, which favors thinner fiber formation. In short-side-chain Aquivion^TM^, at an optimized solvent composition between water and IPA along with 20% Aquivion^TM^ and 1 to 1.5% PEO, nanoribbons were formed after electrospinning. PEO was removed after a washing process, and the mats were densified under high pressure. The electrospun mats showed ionic conductivity similar to that of cast films with the same polymer volume fractions under majority of relative humidity conditions. At lower relative humidity (80% RH), the electrospun mats showed higher ionic conductivity for both Fumion^®^ and Aquivion^TM^, which may have been due to the porosity of the membranes.

Solvent composition is another important factor that can impact nanofiber morphology. Mann-Lahav et al. developed the first Fumion^®^ (FAA-3, a quaternary-ammonium-functionalized-aromatic-Poly(p-phenylene oxide)-based polymer) ionomeric nanofibers and studied the anion conductivity of individual fibers [44]. They investigated how changes in solvent composition and ionomer precursor concentration affected nanofiber morphology, which varied from cylindrical to flat belts and non-flat belts (Figure 2f–i). The water uptake of all the nanofiber mats was much greater than that of solid films due to increased water capillary condensation at fiber crossover points. The Br^−^ anion conductivity for the individual-nanofiber mat was significantly higher than that of the solid films cast from the base ionomer, reaching approximately 30 mS cm^−1^.

**Figure 3:**
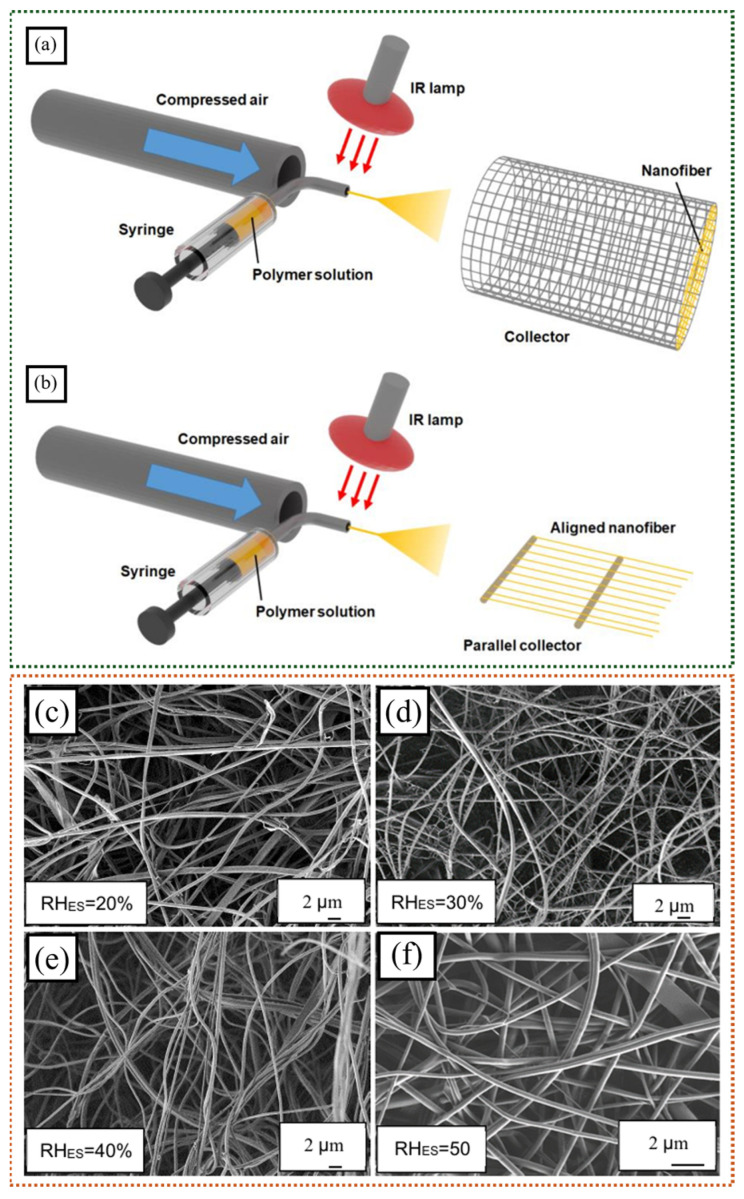
Schematics of (**a**) base SBS setup and (**b**) modified SBS setup for aligned nanofiber spinning (adapted from reference [34], Copyright © 2021 by the authors). SEM images of electrospun ionomer nanofibers at (**c**) 20% RH, (**d**) 30% RH, (**e**) 40% RH, and (**f**) 50% RH (adapted from reference [46], Copyright © 2020 by the authors.).

The processing method employed also plays a crucial part in determining nanofiber morphology. Shinkawa et al. developed a high-throughput method called solution blow spinning (SBS) to produce high-purity Nafion^®^ nanofibers (NFs) with the help of PEO as a spinning aid and carrier polymer matrix (Figure 3a,b) [34]. The nanofibers produced using blow spinning were different from those made via electrospinning. The separation of the hydrophilic and hydrophobic parts for the nanofibers was reduced, but the crystallization of the CF_2_-CF_2_ chains increased. These differences might have been due to the greater speed and different electrical conditions in blow spinning compared to electrospinning [45]. SBS is better for producing large quantities of nanofibers from solutions containing electrolytes. High-quality nanofibers made using these methods can be used in various applications, like fuel cells, catalysts, water electrolysis, electrodialysis, reverse electrodialysis, and capacitive deionization. They can be used as porous mats or combined with other materials to create composite membranes.

Relative humidity (RH) significantly influences the morphology of ionomeric nanofibers, thereby impacting their subsequent properties. Halabi et al. studied the impact of humidity on the morphological and anion-conducting properties of electrospun FAA-3 [46]. An ionomer electrospinning solution was prepared with a fixed concentration of DMF and varying RHs (20–50%). The nanofibers produced at a RH between 20% and 40% exhibited branching, while those prepared at higher RHs showed minimal changes or limited branching (Figure 3c–f). The nanofibers prepared at low RH (20%) demonstrated the highest water uptake and through-plane anion conductivity, with these properties decreasing as RH increased.

## 3. Current Applications of Ionomeric Nanofibers

### 3.1. Ion/Anion Exchange Membranes

Ion/anion exchange membranes (IEMs and AEMs) are essential components in diverse electrochemical applications, including fuel cells, batteries, and water treatment. These membranes selectively transport ions across a barrier, enabling the efficient transfer of charge and mass [47]. Recent advancements in electrospinning technology along with ionomer involvement have progressed the development of IEMs and AEMs with unique properties and enhanced performance [48,49,50,51].

Recent studies have focused on enhancing the properties and performance of IEMs and AEMs by incorporating ionomers and exploring different electrospinning techniques. Roddecha et al. tested the mechanical and ionic properties of electrospun quaternized ammonium polysulfones [48]. The functionalized polysulfone ionomer membranes were prepared via electrospinning polymers containing QAPS and DMF. The electrospun mats were then solvent-welded and filled with a non-conductive elastomer (poly(dimethyl siloxane)) and a curing agent (Sylgard^®^ 184) to create high-density membranes. The use of QAPS-Cl (quaternary ammonium chloride polysulfone), which has quaternary ammonium groups, allowed for the creation of AEMs. The addition of the elastomer enhanced the mechanical properties and robustness of the membranes. The resulting membranes demonstrated high ion conductivity and good mechanical properties, making them strong contenders for application in fuel cells, dialysis, and ion-selective separations.

**Figure 4:**
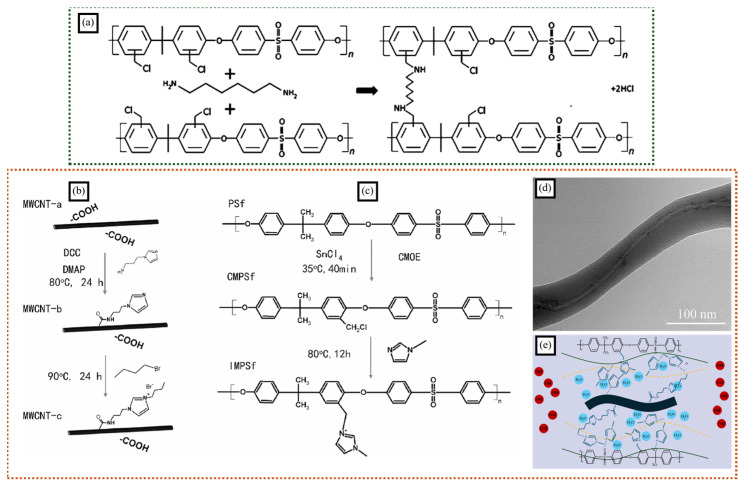
(**a**) Schematic depicting the reaction of chloromethylated polysulfone (CMPSF) with diamine (hexamethylenediamine) towards cross-linked polysulfone (adapted with permission from reference [49], Copyright © 2013 American Chemical Society). Reaction schematic of (**b**) imidazolium-functionalized MWCNTs and (**c**) an imidazolium functionalized polysulfone (IMPSf). (**d**) TEM image of a MWCNT-embedded nanofiber. (**e**) Possible mechanism of co-electrospun FMWCNT/IMPSf composite membrane for hydroxide transport (adapted with permission from reference [51], © 2018 Hydrogen Energy Publications LLC.).

Crosslinking polymers can help further increase the ionic conductivity and mechanical properties of the membrane. Park et al. crosslinked two different electrospun nanofibers containing ionomer fibers to prepare an ion exchange membrane using phase separation morphology [49]. The ionomer fibers (chloromethylated polysulfone, CMPSF) provided high ion exchange capacity (IEC), whereas the non-ion-conducting polymer fibers (Poly(phenylsulfone), PPSU) controlled fiber swelling and enhanced mechanical properties. The crosslinking was carried out at the chloromethyl groups with different diamines in a DMAc/water solvent (Figure 4a). Furthermore, PPSU was used to fill the void of the nanofibrous membrane. At a crosslinking rate of more than 4%, the membrane exhibited a superior IEC than uncrosslinked, water-insoluble quaternary ammonium polysulfone (3.1 vs. 2.5 mmol/g). Crosslinked nanofiber composite membranes demonstrated superior hydroxide ion conductivity (65 mS/cm in room-temperature water) with enhanced swelling (144%) and excellent mechanical properties.

Polyelectrolytes offer a way of creating extremely thin but efficient barriers in membrane technology. The incorporation of polyelectrolytes onto existing NF membranes has significantly improved their development [52]. Seino et al. fabricated polyelectrolyte composite membranes with ion exchange nanofibers (IEX-NFs) and studied the benefits of adding these nanofibers [50]. The polymer matrix is made of Nafion^®^ and poly(vinyl alcohol) (PVA)-based NFs, including poly(vinyl alcohol-b-styrene sulfonic acid) (PVA-b PSS) NFs, which were used as IEX-NFs. The PVA-based NFs were well dispersed in the polymer matrix, forming a 3D network structure. The ionic conductivities of the PVA-b PSS-NF/Nafion^®^ composite membranes were significantly higher than that of the Nafion^®^ membrane, especially in a HCl solution. Electrokinetic measurements revealed that the high density of ion-exchange groups on the NF surface created efficient ion transport pathways within the polymer matrix. Moreover, the mechanical strength of all NF composite membranes was approximately twice that of the membrane without NFs, demonstrating the reinforcing effect of the cross-linked PVA-based NFs.

Incorporating inorganic additives can substantially increase the mechanical strength of polymeric nanofibers. Functionalizing additives with ionic liquids or ionic liquid polymers can enhance hydroxide conductivity, improve compatibility between inorganic and organic components, and optimize particle distribution. The anchoring properties of functional groups at the interface plays a crucial role in these improvements [53]. Among various inorganic additives, multiwalled carbon nanotubes (MWCNTs) are advantageous because they allow easier functionalization and provide advanced properties.

Gong et al. developed co-electrospun inorganic–organic nanofibers consisting of functionalized MWCNTs and polysulfone in order to improve the MWNCTs’ dispersion and enhance the performance of the AEMs [51]. The strong electric field during electrospinning aligns the MWCNTs alongside the axis of the polymer nanofibers, promoting increased dispersion [54]. The high length-to-diameter ratio of the MWCNTs (over 1000) enables excellent synergistic reinforcement with the polymer nanofibers. The imidazolium salt functional groups on the MWCNTs and polysulfone interact to form a better-connected ion transport channel (Figure 4b,c). The involvement of MWCNTs enhances the tensile stress and anti-swelling capability of the nanofiber membrane due to polymer fibration and the innate high modulus of MWCNTs. The combined effects of polymer fibration and aligned MWCNTs improve ion–ion interactions and lead to discrete hydrophilic/hydrophobic micro-separation. This enhanced micro-separation results in a lower-energy barrier for hydroxide transport and a favorably connected pathway, significantly increasing hydroxide conductivity (Figure 4d,e). Composite electrospun membranes incorporating MWCNTs exhibit significantly higher single-cell performance compared to AEMs without MWCNTs. The highest power density of the composite membranes (102.5 mW/cm^2^) is about 1.2 and 11.6 times more than that of base polymer nanofiber membrane and cast AEMs without MWCNTs, respectively.

### 3.2. Proton Exchange Membrane

**Figure 5:**
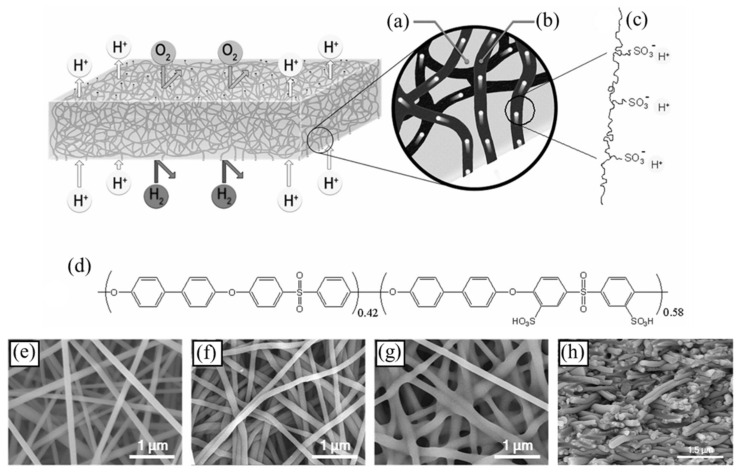
Schematic of nanofiber network composite for PEM: (**a**) polymer matrix restricting the swelling of the nanofibers and enhancing mechanical properties, (**b**) water-swollen sulfonated cation exchange polymer fiber network. Polymer (**c**) schematic and (**d**) chemical structure form. Transformation of electrospun fiber mat: (**e**) initial electrospun mat, (**f**) mat after fiber densification, (**g**) mat after fiber welding, and (**h**) mat after being impregnated with inert polymer (adapted with permission from [55], Copyright © 2008 American Chemical Society).

Proton exchange membranes (PEMs), like IEMs and AEMs, have been developed using ionomeric nanofibers. Instead of ions (or targeted anions), PEMs target the transport of protons (or H^+^ ions) through them. Choi et al. directly prepared a mat of ionomeric nanofibers by electrospinning high-molecular-weight sulfonated poly(arylene ether sulfone) (sPAES) in dimethylacetamide [55]. The sPAES was first prepared via a nucleophilic aromatic substitution condensation reaction of three different monomers (4,4′-Dichlorodiphenyl sulfone (DCDPS), 4,4′-biphenol (BP), and 3,3′-Disulfonated DCDPS (ds-DCDPS)). The nanofiber mats were later densified under pressure and welded by exposing to DMF vapor to increase the number of interconnecting protonic pathways, and the voids were filled with an inert polymer (Norland Optical Adhesive, NOA-63). The prepared mat shows enhanced isotropic proton conductivity at different volume fractions, improved mechanical property due to densified interconnections, reduced swelling and enhanced gas barrier properties due to inert polymer filling nanofiber mat voids (Figure 5a–h).

Zhang et al. fabricated electrodes with electrospun nanofiber catalyst layer (NFCL) cathodes, achieving high power densities at various platinum (Pt) loadings [56]. The crucial factors contributing to improved mass transport and proton transfer include Knudsen diffusion resistance in pores and oxygen permeation resistance in ionomer films and nanofiber structures. The authors evaluated the electrochemical impedance spectra (EIS) of the materials both in situ and ex situ. The results indicated that the low proton transfer resistance (R_H+_) observed could be attributed to the well-formed ionomer films and the increased proton conductivity of the ionomer fibers. A PFSA ionomer was used along with poly(acrylic acid) (PAA) as a carrier and sacrificial nanofiber polymer to prepare NFCL. The results of the tests were compared with those obtained from a conventionally available particle-stack catalyst layer (PCL) with PFSA in a similar volume ratio. Power density performance for the NFCL (1235 mW cm^−2^) was 9.4% higher than that for the PCL (1128 mWcm^−2^) at 2 A cm^−2^ with a 0.15 mg_Pt_ cm^−2^ cathode loading. The authors investigated the impact of the microstructure of the ionomer film and free ionomer fibers on the interaction between ionomers and Pt, ionomer coverage, and proton accessibility. They discovered that the uniformly distributed ionomers with defect areas or ultra-thin sites created due to the removal of the PAA carrier polymer led to more three-phase reaction interfaces.

Additionally, the electric fields during electrospinning extended the ionomer films, resulting in thinner films that facilitated easier O_2_ diffusion. Moreover, the presence of ultra-thin ionomer fibers spread within the longer, homogeneous catalyst/ionomer fibers significantly the proton transfer. Overall, these findings highlight the crucial role of ionomer film microstructures and free ionomer fibers in enhancing the performance of the catalyst.

As discussed above, changing the method used to prepare nanofibers can change their properties. Onuki et al. presented a different technique for preparing ionomer composite membranes through highly scalable blow-spun fiber formation for enhancing proton conductivity and mechanical properties [57]. PFSA thin fibers were created using the blow-spinning technique [34,58]. This method was chosen because the inherent electrical charges in PFSA dispersions can interfere with the traditional electrospinning process [59]. Aquivion^TM^, a short-side-chain PFSA, was used as both the main polymer material and the structure for the thin fibers. The nanofibrous mat was hot-pressed to increase stability and fiber connectivity. The hot-pressed PFSA nanofiber network served as an interconnected framework within the membranes, which improved proton transport and enhanced mechanical properties. A composite membrane containing 15 wt% PFSA thin fibers exhibited better fuel cell (FC) performance than the original PFSA membrane under both low-temperature with high-humidity and high-temperature with low-humidity conditions. The maximum power density (P_max_) values increased by 14–19%.

**Figure 6:**
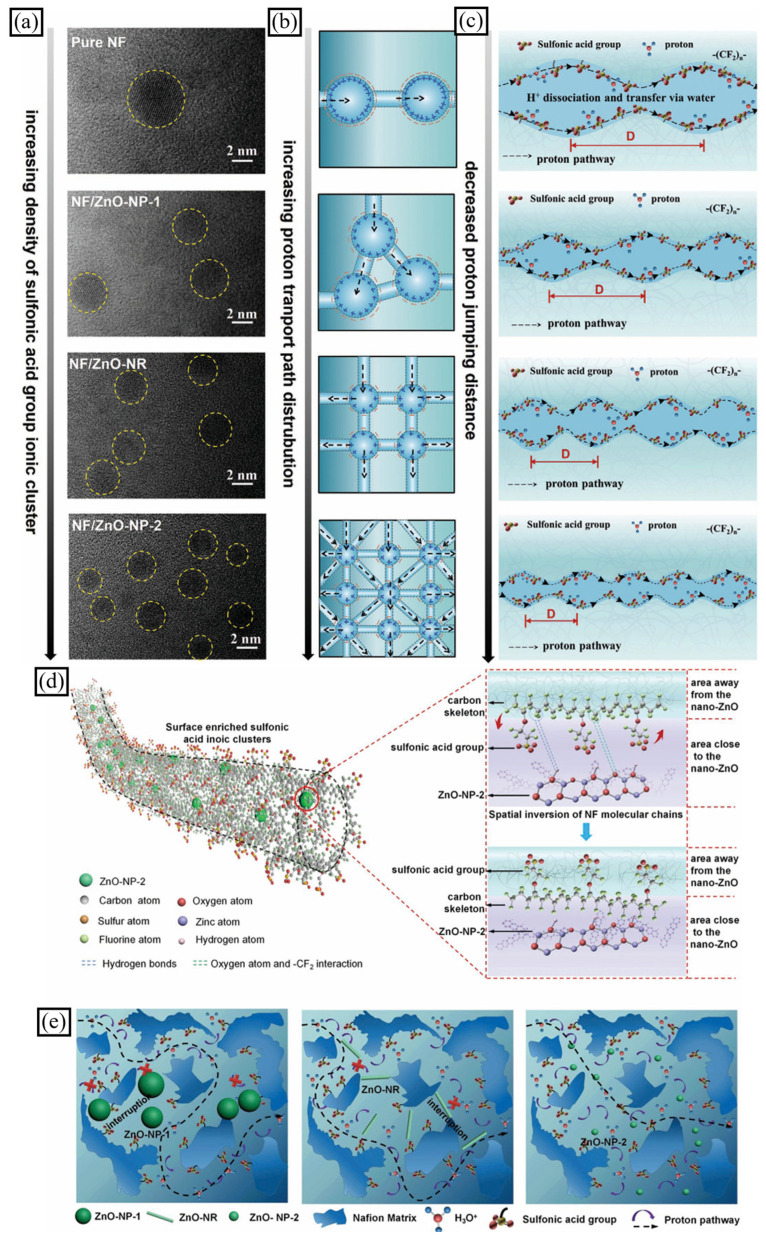
The nano-physical distribution and geometry of sulfonic acid ionic clusters were investigated. (**a**) High-resolution TEM image. Graphical representation of (**b**) path distribution and (**c**) jump trajectory of proton transport in sulfonic acid ionic clusters (sulfonic acid ionic clusters are represented by yellow circles). (**d**) Chemical structure and molecular model of NF/ZnO nanofibers during intermolecular electrostatic interactions and hydrogen bonding for nano-ZnO and -CF_2_ groups. (**e**) Schematic for nano-ZnO morphology and size effects on the pathway of proton transport within the fiber region (adapted with permission from [60], Copyright © 2020 WILEY-VCH Verlag GmbH & Co. KGaA, Weinheim.).

Metal oxide nanoparticles can interact with the side chains of ionomers and cause changes in the overall nanofiber properties. Jin et al. prepared a composite of Nafion^®^ nanofibers (NF) doped with zinc oxide nanofillers (nano-ZnO) for anisotropic proton transport [60]. The sturdy electrostatic interaction between the nano-ZnO and the -CF_2_ groups of the NF backbones induced a spatial inversion of sulfonic acid groups within the NF molecules, leading to high-density ionic clusters and long-range continuous ionic nanochannels on the NF/ZnO nanofibers’ surface (Figure 6). This resulted in high proton conductivity (up to 0.253 S cm^−1^) and power density (115.72 mW cm^−2^) in an application of DMFC (Direct methanol fuel cell), for the NF-based PEMs. The larger size of the nano-ZnO in NF/ZnO-NP-2 nanofibers slightly hindered the continuity of the nanochannels, leading to increased tortuosity and reduced anisotropy of proton transport (Figure 6e). However, these nanofibers still demonstrated high conductivity and power density in a direct methanol fuel cell (DMFC) application. The single-step electrospinning preparation technique and economical nano-ZnO make NF/ZnO-NP-2 nanofibers ideal for industrial use as PEMs.

**Figure 7:**
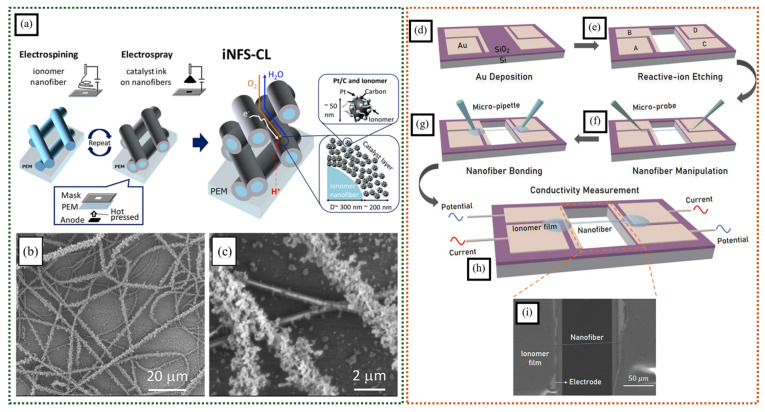
(**a**) Fabrication schematic and structure of ionomer nanofiber scaffolding catalyst layer. (**b**,**c**) SEM figures of iNFS-CL (one cycle) (adapted with permission from [61], © 2020 Elsevier B.V.). The process of fabricating a micro-electrode with a single ionomer nanofiber; (**d**) deposition of Au on SiO_2_; (**e**) etching of the substrate; (**f**) immobilization of the nanofiber on a micro-electrode; (**g**) attachment of the nanofiber and micro-electrode using ionomer solution droplets; (**h**) drying of the ionomer droplets; (**i**) SEM image of the ionomeric nanofiber attached to microelectrode via ionomer solution droplets (adapted with permission from [62], © 2022 Elsevier Ltd.).

Electrospinning can be combined with other methods, like electrospraying, to facilitate a more compact interaction between the nanofiber membrane and catalyst ink. Yoshino et al. created catalyst layers with ionomeric nanofiber scaffolds by alternating electrospinning Nafion^®^ nanofibers and electrospraying a catalyst ink containing Pt/C and additional Nafion^®^ (Figure 7a–c) [61]. They studied how different ionomer loadings and distributions within the catalyst layer affected cell performance under various humidity conditions. The iNFS-CLs showed improved resistance to changes in humidity, which was attributed to better water and oxygen transport in the catalyst layer through the continuous pores. The preparation method used also reduced the ionomer percentage in the catalyst ink, minimizing poisoning effects induced by the anionic groups. Although expected, there was no decrease in the tortuosity for proton conductivity in the catalyst layer, possibly due to the lack of crosslinking between the nanofibers.

A non-uniform nanofiber membrane may show diverse conductivity in different planes. Wang et al. developed a technique to measure the proton conductivity of individual ionomer nanofibers [62]. They used a specialized microelectrode chip with gold probes to hold the nanofiber in place and prevent interference via contact resistance (Figure 7d–i). The nanofiber was connected to the probes using the same solution it was made from, creating a strong bond. This method was more effective than using electron beams to create the connection. To measure the proton conductivity of the pure ionomer nanofiber without interference from the carrier polymer, a pre-treatment was used. This study found that the presence of the carrier polymer, PEO, reduced proton conductivity. The researchers also examined how RH affects proton conductivity and discovered that ionomer nanofibers are more sensitive to RH changes than ionomer films due to their large surface areas. This measurement method can be useful for designing and improving catalyst layers or polymer electrolyte membranes made with ionomer nanofibers. It can also be used with different types of ionic conductive nanofibers or films.

Crosslinking nanofibers with the matrix can also enhance mechanical and ion exchange properties. Han et al. developed a high-performance fuel cell membrane by embedding sulfonated poly (ether ketone) (SPEEK) nanofibers within a cross-linked SPEEK matrix [63]. This innovative design enhanced the membrane’s stability, strength, and conductivity, leading to improved fuel cell performance at elevated temperatures and under high humidity. During thermal treatment, a cross-linked matrix formed, enhancing the membrane’s structural integrity and water resistance. Simultaneously, the non-cross-linked nanofibers remained intact, forming long-range proton transport channels that facilitated efficient proton conduction. The C-SP90/SP90NF membrane, with a 90% sulfonation degree, achieved a peak power density of 485 mW/cm^2^ at 80 °C and 100% relative humidity, outperforming the cross-linked SPEEK matrix by 1.45 times. This significant improvement demonstrates the potential of this novel membrane design for use in advanced fuel cell applications. Table 3 shows a comparison of a few of the ionomeric nanofibers with respect to their ion exchange membrane capability.

### 3.3. Tissue Engineering

Ionomer-based materials have emerged as capable contenders for tissue-engineering applications because of their unique properties, including biocompatibility, tunable mechanical properties, and the possibility for the incorporation of functional groups for targeted biological interactions. Ionomeric nanofibers offer a means of creating highly porous and aligned scaffolds that can imitate the extracellular matrix (ECM) and promote cell growth and differentiation [75,76].

**Figure 8:**
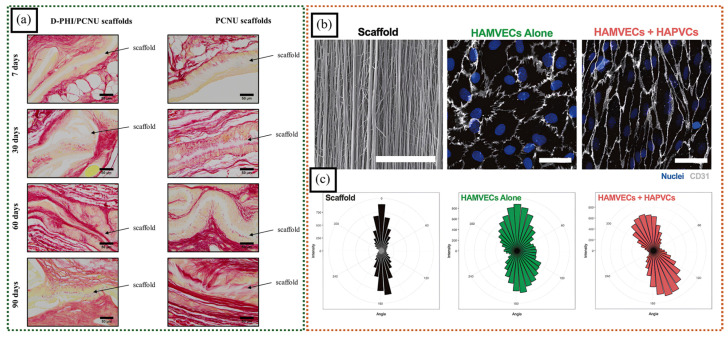
(**a**) Histological analysis of D-PHI/PCNU and PCNU scaffolds, stained with Picrosirius red, revealed tissue integration over time. The scaffolds, stained pale yellow, were found to integrate with surrounding tissue, stained dark yellow, and collagen fibers, stained red (adapted with permission from [75], Copyright © 2019 Acta Materialia Inc.). (**b**) These SEM images visualize the three-dimensional topography of the scaffold. Immunofluorescent staining techniques were employed to examine the morphology of the HAMVECs. Scale = 50 μm. (**c**) Rose plots were used to illustrate the average distribution and orientation of the cells (adapted with permission from [76], Copyright © 2023 Acta Materialia Inc.).

Ionomers can induce better biocompatibility for cell growth and adhesion. Chan et al. co-electrospun degradable polar hydrophobic ionic polyurethane (D-PHI) and high-molecular-weight degradable linear polycarbonate polyurethane (PCNU) to create aligned nanofibrous scaffolds for tissue engineering [75]. These scaffolds exhibited higher vinyl conversion, higher surface energy, and decreased stiffness compared to PCNU scaffolds. In vivo studies revealed slow degradation, excellent tissue integration, and promotion of cell growth (Figure 8a). The distinctive properties of D-PHI in these scaffolds make them capable contenders for various tissue-engineering applications.

The diverse functional groups provided by ionomers in membranes can aid in the selective separation of cells. Antonyshyn et al. prepared an ionomeric polyurethane nanofiber scaffold designed to be applied as a pseudo-basement nanofibrous membrane in the physical separation of endothelial cells from perivascular cells [76]. Human-adipose-tissue-derived perivascular cells (HAPVCs), when cultured on the opposite side of the scaffold (due to enhanced adhesion via the presence of an ionomer) with respect to human-adipose-tissue-derived microvascular endothelial cells (HAMVECs), induced the adhesion, elongation, and alignment of HAMVECs, leading to a more mature and stable endothelial lining (Figure 8b). In this study, ionomeric polyurethane used was chosen for its hemocompatibility and non-immunogenicity, which make it suitable for use in applications involving contact with blood and living tissue.

### 3.4. Membrane Distillation

Nanofibers and ionomers are promising materials with potential applications in membrane distillation. Nanofibers, with their enhanced porosity and surface area, can improve heat and mass transfer, offering a more efficient distillation process [77,78]. Ionomers, on the other hand, can regulate water molecules’ transportation through membranes, improving water flux [79,80]. Ionomeric nanofibers combine these advantages with fewer fabrication steps and reduced costs. They offer high porosity and surface area and enhanced water transport, making them promising materials for use in various distillation techniques [81,82,83,84,85,86].

**Figure 9:**
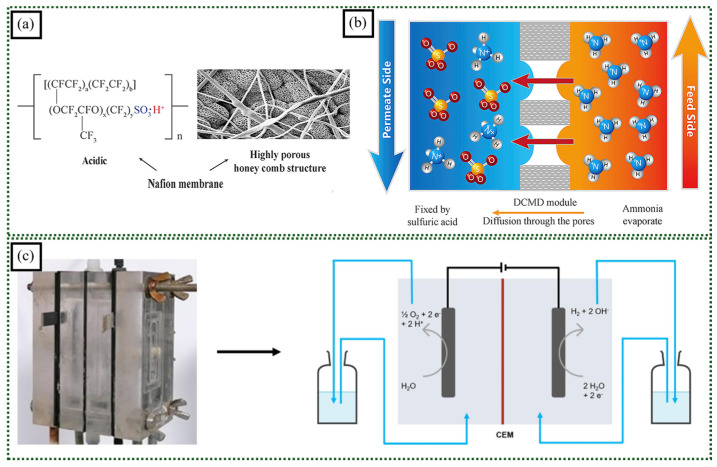
(**a**) Chemical structure and SEM image of highly porous honeycomb-structured Nafion/PVDF membrane (colored segment representing the sulfonic acid of the ionomer). (**b**) Schematic diagram of the ammonia recovery process via DCMD (adapted with permission from [82], Copyright © 2019 Elsevier B.V.). (**c**) Lab-scale electrochemical setup made from plexiglass with two compartments separated by CEM (adapted with permission from [85], Copyright © 2022 Elsevier B.V.).

Typically, it is challenging to directly electrospin PFSA polymers into nanofibers. To overcome this limitation, researchers often incorporate low concentrations of PFSA into other electrospinnable polymers to facilitate the formation of electrospun mats. Guo et al. prepared an ammonia recovery membrane with a highly porous honeycomb nanostructure consisting of a Nafion^®^-incorporated PVDF membrane [82]. Nafion^®^ membranes significantly improve ammonia recovery efficiency in direct-contact membrane distillation (DCMD) (Figure 9a,b). Their unique properties, including high proton conductivity and strong water affinity, enhance mass transfer by facilitating the diffusion of ammonia molecules through the membrane’s porous structure. Additionally, Nafion’s^®^ hydrophilic nature selectively attracts and transports ammonia molecules, rejecting other solutes. The smooth surfaces of Nafion^®^ membranes minimize fouling, ensuring sustained performance. By incorporating Nafion^®^ into the membrane, the authors achieved significantly higher ammonia recovery rates compared to those achieved using traditional PVDF membranes.

Swanckaert et al. revealed the potential of sulfonated silica-based nanofiber cation exchange membranes (CEMs) for enhancing electrochemical water treatment processes [85]. By directly electrospinning a sol–gel solution containing tetraethyl orthosilicate (TEOS) and 3-mercaptopropyl triethoxysilane (MPTES), the researchers successfully produced nanofiber membranes with a high ion-exchange capacity. The resulting membranes exhibited excellent chemical resistance, particularly against strong acids and chlorine, surpassing the performance of commercial CEMs (Figure 9c). Additionally, the nanofiber CEM demonstrated remarkable self-cleaning properties, effectively removing fouling deposits without the need for harsh chemical cleaning. This unique feature significantly reduces maintenance costs and extends the membrane’s lifespan.

The incorporation of nanomaterials into polymer matrices can significantly alter the morphology and properties of the resulting nanofibers, increasing their potential applications. Talukder et al. prepared a superhydrophobic nanofiber membrane of SPES containing a bead-like structure formed by MWCNTs for enhanced membrane distillation [83]. The incorporation of hydrophobic engineered nanomaterials, specifically S-MWCNTs, into an SPES polymer matrix enhanced membrane distillation by controlling water swelling and mechanical properties. The incorporation of S-MWCNTs significantly improved water flux and salt rejection compared to bare SPES membranes. The bead-like structures that formed on the membrane surface due to the incorporation of S-MWCNTs increased hydrophobicity and reduced wetting, leading to higher water vapor flux. These beads created a rough surface with a higher contact angle, leading to increased hydrophobicity and reduced wetting. Additionally, the S-MWCNTs enhanced the mechanical strength and thermal stability of the membrane.

Multi-ionic polymers, such as zwitterionic polymers, can be employed in advanced separation processes like mixed solvent distillation. Chiao et al. developed a novel bilayer electrospun zwitterionic membrane to allow enhanced membrane distillation performance in treating hydraulic-fracturing-produced water [84]. The membrane’s unique design, incorporating an omniphobic surface facing the permeate stream and a hydrophilic surface facing the feed stream, significantly improved its fouling resistance and wetting resistance. The omniphobic surface, created through the growth of silica nanoparticles and subsequent silanization, minimized wetting by low-surface-energy compounds. The hydrophilic surface, grafted with a zwitterionic polymer, repelled oil and other organic contaminants, further enhancing the membrane’s antifouling properties. This bilayer design resulted in improved membrane stability, flux, and salt rejection, even when challenged with challenging feed solutions containing high concentrations of salts, organic compounds, and surfactants.

### 3.5. Metal Ion Removal

Nanofiber membranes can also function as promising materials for the removal of metal ions from aqueous solutions due to their large surface areas, high porosity, and tunable properties. By incorporating ionomers into these membranes, it is possible to enhance their selectivity, adsorption capacity, and stability [87,88,89,90,91,92]. Zhao et al. fabricated PFSA ionomer/poly(N-vinylpyrrolidone) (PFSA/PVP) nanofiber membranes for metal ion removal and enhanced proton conductivity [92]. A nanofibrous membrane was prepared by electrospinning the polymer mixture in DMF solvent after degassing and under controlled humidity. Cross-linking in the membrane was improved by adding different concentrations of 4,4′-diazostilbene-2,2′-disulfonic acid disodium salt (DAS). Applying heat treatment or UV radiation to DAS generates nitrogen (N_2_) and highly reactive nitrene intermediates. These intermediates then react with PVP in an oxygen-free environment, forming cross-linked networks [93].

The stability of the fiber membranes in water improved as the amount of PFSA increased. Adding DAS made the PFSA/PVP electrospinning process easier, resulting in the nanofiber membranes having a uniform diameter of 100 nm. After heat treatment, the water stability of the DAS-containing PFSA/PVP nanofiber mats significantly increased. These water-stable membranes effectively removed metal ions from water, as demonstrated by the rapid adsorption of Cu^2+^ and Ca^2+^ ions, with maximum adsorption capacities of 43.10 and 22.37 mg/g, respectively.

Sulfonation can also be replaced by the use of larger ionic materials with inbuilt ionic groups. Zhen et al. prepared an ionic-liquid-grafted polyethersulfone (PES-g-IL) nanofibrous membrane for dye absorbance, heavy metal removal, and antibacterial property bestowment [89]. These PES-g-IL nanofibrous membranes are ionomers due to the presence of ionic liquid (IL) groups grafted onto the PES backbone. These ionic groups provide the material with ion exchange properties, enabling it to effectively adsorb both anionic dyes and cationic heavy metal ions. The electrostatic interactions between the charged groups on the membrane and the ions in solution contribute to the high adsorption capacity and selectivity of this material. Various ionomeric nanofibers have been compared regarding their heavy metal ion adsorption capability in Table 4.

### 3.6. Battery and Energy Storage

Battery and energy storage technologies are rapidly evolving to meet the growing demand for sustainable and efficient energy solutions. Redox flow batteries, lithium-ion batteries, and solid-state lithium metal batteries are among the most promising technologies in this field. Ionomers play a crucial role in these technologies. They are used as electrolytes, separators, and binders, enhancing the performance and durability of batteries [29,94,95,96]. Ionomeric nanofibers offer unique advantages due to their high surface area, excellent mechanical properties, and tunable ionic conductivity. By incorporating ionomeric nanofibers into battery components, researchers aim to develop advanced batteries with improved energy density, power density, and cycle life.

Saadi et al. developed high-performance hydrogen electrodes for hydrogen bromine redox flow batteries (HBRFBs) using electrospun Nafion^®^-PEO core–shell nanofibers [97]. By incorporating a core–shell nanofiber structure with a hydrophobic Nafion coating, they were able to significantly reduce the platinum loading while maintaining excellent performance and durability. The hydrophobic Nafion coating protected the platinum catalyst from corrosion by bromine species, extending the battery’s lifespan.

**Figure 10:**
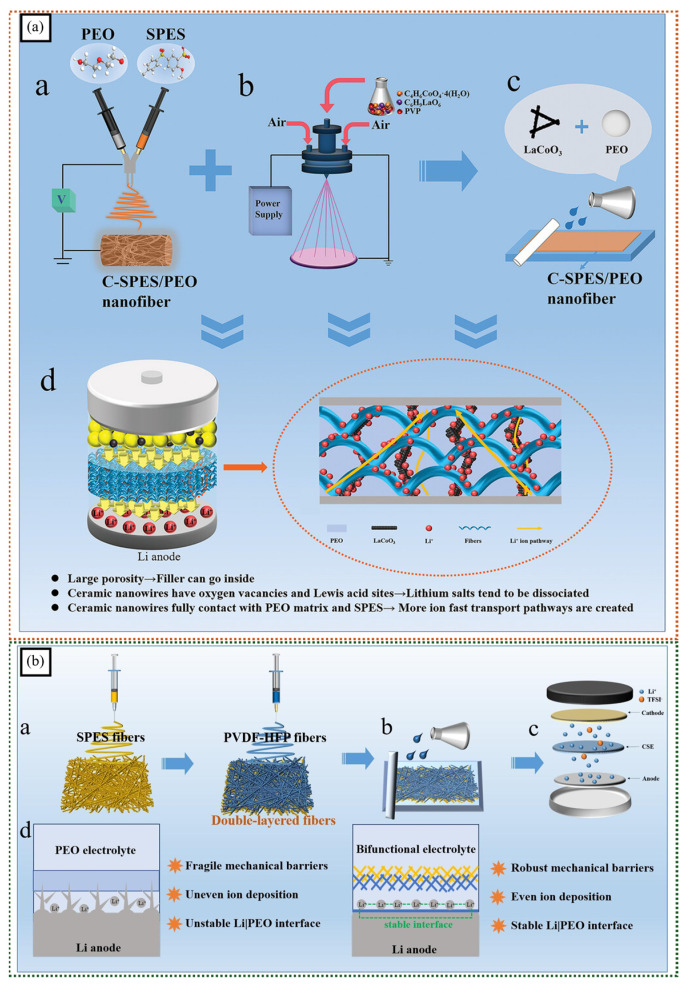
(**a**) A schematic of fabrication of (a) 3D crimped nanofiber membranes, (b) LaCoO_3_ nanowires, and (c) C-SPES-PEO-LaCoO_3_ composite solid electrolytes. (d) Promotion effect of C-SPES-PEO-LaCoO_3_ electrolyte on lithium ion (adapted with permission from [98], copyright © 2023 Wiley-VCH GmbH). (**b**) Schematic of the preparation of (a) double-layer SPES-PVDF-HFP nanofiber membrane, (b) composite solid electrolyte, and (c) all-solid-state lithium battery. (d) Working principle of electrolyte (adapted with permission from [99], Copyright © 2024 Elsevier B.V.).

Yang et al. introduced a novel approach to solid-state electrolyte design, utilizing a 3D crimped sulfonated polyethersulfone-polyethylene oxide (C-SPES/PEO) nanofiber membrane and long-range LaCoO_3_ nanowires [98]. The ionomeric SPES/PEO nanofibers create a porous, interconnected network that facilitates ion transport and enhances mechanical strength. The LaCoO_3_ nanowires, dispersed within the nanofiber matrix, further improve ionic conductivity by providing additional ion conduction pathways and facilitating the dissociation of lithium salts. The unique combination of these components leads to a composite electrolyte with exceptional properties, including high ionic conductivity, excellent mechanical strength, and enhanced electrochemical stability (Figure 10a). This innovative approach has the potential to revolutionize solid-state battery technology, enabling the development of high-performance, long-lasting energy storage devices.

Building on this work, Yang et al. developed a high-performance solid-state electrolyte for lithium metal batteries [100]. By combining dendritic sulfonated polyethersulfone (SPES) nanofibers with lanthanum cobaltate (LaCoO_3_) nanowires, the researchers created a composite electrolyte with enhanced mechanical strength, high ionic conductivity, and excellent electrochemical stability. The ionomeric SPES nanofibers provided a 3D network structure that facilitated ion transport and improved the mechanical properties of the electrolyte. Additionally, the LaCoO_3_ nanowires acted as effective lithium-ion conductors and contributed to the stability of the lithium metal anode. The ionomeric SPES nanofibers provided a 3D network structure that facilitated ion transport, while the LaCoO_3_ nanowires acted as effective lithium-ion conductors, improving the overall performance of the lithium metal batteries.

Furthermore, Yang et al. prepared a double-layer SPES/poly(vinylidene fluoride-co-hexafluoropropylene) (SPES/PVDF-HFP) nanofiber membrane to construct a dual functional composite solid electrolyte for all-solid-state lithium metal batteries [99]. The ionomeric SPES and PVDF-HFP nanofibers play a crucial role in enhancing the performance of the solid-state electrolyte. The SPES nanofibers act as ion-conducting channels, facilitating lithium ion transport through the electrolyte. The PVDF-HFP nanofibers, on the other hand, contribute to the formation of a stable solid electrolyte interphase (SEI) layer on the lithium metal anode, preventing dendrite growth and improving the long-term cycling stability of the battery (Figure 10b). The combined effect of these ionomeric nanofibers results in a composite electrolyte with high ionic conductivity, excellent mechanical strength, and superior electrochemical stability.

### 3.7. Analyte Monitoring and Sensing

Ionomeric polymers, particularly sulfonated polymers like SPEEK, have shown remarkable potential in the development of advanced sensing technologies [101,102]. Their unique properties, such as ion conductivity and sensitivity to environmental stimuli, make them ideal candidates for various sensing applications. By incorporating ionomers into nanofiber structures, researchers have achieved significant improvements in sensitivity, response time, and selectivity.

Wang et al. prepared an adsorbent/conducting polymer one-dimensional core–shell nanostructure: SPEEK/PPy (polypyrrole) core–shell nanofibers for ammonia (NH_3_) gas sensing [103]. The ionomeric polymer, SPEEK, played a crucial role in enhancing the sensitivity and selectivity of the gas sensor. By incorporating SPEEK into the core–shell nanofiber structure, the researchers were able to improve the adsorption of NH_3_ molecules, leading to a significant increase in the sensor’s response. The sulfonic acid groups in SPEEK facilitated interaction with NH_3_ molecules, promoting their diffusion through the PPy shell and enhancing the overall sensing performance (up to 20 ppb).

Choi et al. prepared a wireless and flexible humidity sensor with SPEEK nanofibers [104]. The sulfonic acid groups on the SPEEK chains played a crucial role in the humidity-sensing mechanism. As the relative humidity increased, water molecules were absorbed by the sulfonic acid groups, leading to an increase in proton conductivity. This change in conductivity was directly reflected in the electrical resistance and capacitance of the sensor, enabling accurate and sensitive humidity detection. The high surface area and porous structure of the nanofibers facilitated efficient water vapor adsorption and desorption, resulting in fast response (75 s) and recovery times.

**Figure 11:**
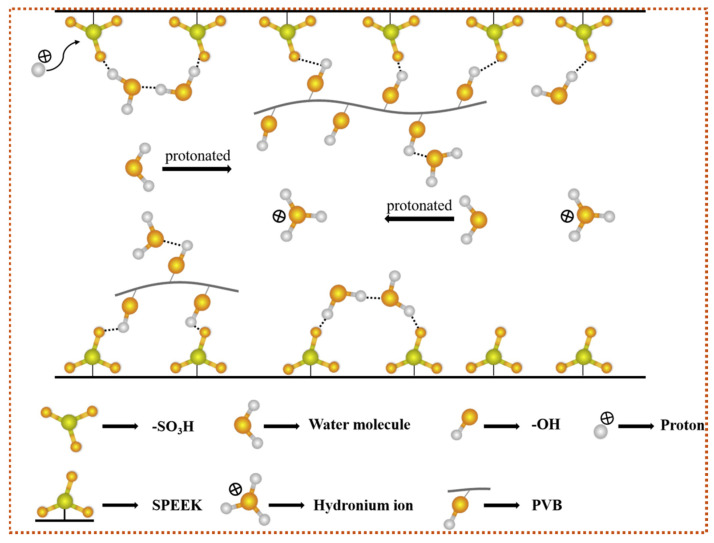
A schematic of the theorized mechanism of the SPEEK/PVB composite nanofiber membrane. (Adapted with permission from [105], Copyright © 2020 Elsevier B.V.).

Li et al. fabricated a breath-monitoring and non-contact humidity sensor based on SPEEK/PVB (polyvinyl butyral) nanofibers [105]. The SPEEK ionomer, when electrospun into nanofibers, exhibited superior humidity-sensing properties compared to its thin-film counterpart. The porous structure of the nanofibers facilitated rapid water vapor diffusion and adsorption, leading to faster response and recovery times. Furthermore, the incorporation of PVB into the SPEEK nanofibers further improved sensing performance. The hydrophilic nature of PVB, along with its hydrogen-bonding interactions with SPEEK, contributed to increasing water absorption and enhancing proton conductivity (Figure 11). This resulted in a significant reduction in hysteresis (2.68%), a faster response time (<1 s), and a quick recovery time (5 s).

## 4. Conclusions and Outlook

In conclusion, this mini review has thoroughly examined the developments in PFSA ionomeric nanofibers, emphasizing their special qualities, production processes, and possible uses. A prospective platform for developing functional materials with specific qualities, such as increased mechanical strength, better processability, and customized functional characteristics, is provided by ionomeric nanofibers. Researchers can continue to broaden the scope of this discipline and create new materials with a variety of uses by comprehending the factors influencing the shape of ionomeric nanofibers and investigating creative production methods.

Future studies should concentrate on a few crucial areas in order to further develop the field of ionomeric nanofibers. Certain qualities including porosity, surface area, and mechanical strength can be improved by creating hierarchical nanostructures, such as hollow or core–shell structures. Nanofiber consistency and quality may be increased by optimizing the electrospinning process using cutting-edge process control methods, unique solvent systems, and creative nozzle designs. For industrial applications, increasing output through roll-to-roll processing, continuous electrospinning, and 3D-printing integration is crucial. Combining ionomeric nanofibers with other materials and functionalizing them with specific chemical groups can create multifunctional materials with tailored properties. Commercialization requires addressing issues relating to, e.g., long-term stability and cost reduction. Researchers can unleash the full potential of ionomeric nanofibers and spur innovation across a range of industries by taking advantage of these opportunities.

## Figures and Tables

**Table 1:** Advantages and disadvantages of nanofibers.

Advantages	Disadvantages
Versatility Nanofibers can be made from various materials, offering customizable properties for various applications.	In situ deposition Directly depositing nanofibers onto specific substrates can be challenging.
High surface area Their nanoscale size provides a large surface area, ideal for functions like sensing and filtration.	Efficiency Electrospinning can result in low yields and require high voltages.
Functionalization Chemical modifications can easily be made to nanofibers to enhance their specific properties.	Scaling Producing nanofibers with desired properties on a large scale can be difficult.
Combinability Different materials can be combined to create nanofibers with tailored characteristics.	Material limitations Aqueous solutions and biomaterials can pose challenges for electrospinning.
Accessibility Electrospinning setups are relatively inexpensive, making nanofiber production accessible.	Uniformity Achieving consistent thickness and electrical dispersion can be problematic, especially with conductive materials.
Ease of learning The electrospinning technique is straightforward.	
Versatile deposition Nanofibers can be deposited onto various surfaces, including metals, glass, and water.	
Structural diversity Electrospinning allows for the creation of various nanofiber structures.	
Scalability Commercial systems can produce nanofibers in large quantities.	
Commercial applications Many products currently on the market utilize nanofiber technology.	

**Table 2:** Advantages and disadvantages of ionomers.

Advantages	Disadvantages
Enhanced mechanical properties Ionomers’ toughness, moduli, and tensile strength may all be greatly enhanced by the ionic interactions between their basic and acidic groups.	Limited processability Ionic interactions between the polymer chains can make processing ionomers challenging, particularly at high temperatures.
Improved adhesion Ionomers can adhere well to a variety of substrates, including metals, ceramics, and plastics, due to their ability to form ionic bonds with a substrate.	Potential for phase separation Ionomers can undergo phase separation, leading to a loss of mechanical properties and other undesirable effects.
Enhanced barrier properties Ionomers’ thick, cross-linked structure can offer superior barrier qualities, which makes them valuable for use in membranes, adhesives, and coatings.	Sensitivity to moisture Some ionomers can be sensitive to moisture, which can affect their properties and performance.
Improved biocompatibility According to research, certain ionomers are biocompatible, which qualifies them for use in biomedical and medical settings.	Higher cost Ionomers can be more expensive to produce than conventional polymers due to the complexity of their synthesis and processing.
Tailored properties By changing the content and structure of a polymer, ionomers’ characteristics may be changed, opening up a variety of avenues.	

**Table 3:** Comparison of different ionomeric nanofibers regarding their ion/proton exchange capability and conductivity.

Nanofibers	Ionic Group	Backbone Chain	IEC (mmol/g)	Water Uptake (%)	Ion Conductivity (S cm^−1^)	Relative Humidity (%RH)	Temperature (°C)	Ref.
Nafion	–SO_3_H	PTFE	–	20–35	0.055–0.06	100	25	[64]
Nafion	–SO_3_H	PTFE	0.9	10–20	0.06–0.08	100	RT	[65]
3M^®^ (EW 733 and 825)	–SO_3_H	PTFE	–	10–50	0.007–0.16	80	80	[66]
3M^®^ (EW 825)—sPOSS	–SO_3_H	PTFE/POSS	–	–	0.498	90	120	[67]
SPEEK	–SO_3_H	PEEK	1.84–2.62	76–96	~0.02–0.1	50–90	80	[68]
SPEEK	–SO_3_H/–NR_4_^+^	PEEK	0.5–0.9	60–85	0.08–0.12	100	25	[69]
SPAES	–SO_3_H	PAES	0.3–2	20–180	0.01–0.12	–	25	[55]
SPAES/SPOSS	–SO_3_H	PAES/POSS	2.1	90	0.1	95	30	[70]
QPPNF	–NR_4_^+^	PPO	0.4–1.4	43–92	11–89	–	80	[71]
sPSU	–SO_3_H/–NR_4_^+^	PSU	1.2–1.3	35–65	0.08–0.26	100	80	[72]
CMPSF	–CH_2_Cl	PSU	1.7–2	144	0.0065	100	23	[49]
QAPS-OH	–NR_4_^+^	PSU	0.6–1.7	–	0.011–0.06	90	80	[48]
sPI	–SO_3_H	PI	1.4	22–29	0.03–0.1	98	80	[73]
sPI	–SO_3_H	PI	1.6	38–42	0.2–0.3	98	90	[74]
PVA-b PSS/Nafion	–SO_3_H	PVA/PSS/PTFE	1.02–1.07	40–44	53–63	–	25	[50]

PTFE—polytetrafluoroethylene, sPOSS—sulfonated polyhedral oligomeric silsesquioxane, POSS—polyhedral oligomeric silsesquioxane, PEEK—poly(ether ether ketone), PAES—poly(arylene ether sulfone), QPPNF—quaternized poly(2,6-dimethyl-1,4-phenylene oxide) nanofiber, sPSU—sulfonated polysulfone, PSU—polysulfone, CMPSF—chloromethylated polysulfone, sPI—sulfonated co-polyimide, and PI—polyimide.

**Table 4:** Comparison of different ionomeric nanofibers regarding their heavy metal adsorption capability.

Nanofibers	Ionic Group	Backbone Chain	Heavy Metal Ion	Initial Conc. (mg/L)	Optimum pH	Adsorption Capacity (mg/g)	Ref.
PES-g-IL	imidazolium cation	PES	Cd^2+^	650	6	75	[89]
Nafion	–SO_3_H	PVA	Cu^2+^, Cr^3+^, Co^2+^, As^3+^	100	5.9	59.1, 42.5, 24.7, 22.7	[88]
SPES	–SO_3_H	PES	Pb^2+^, Cd^2+^, Cr^4+^	150	4, 4, 2	656.42, 315.55, 418.86	[87]
SPES/PES	–SO_3_H	PES	Pb^2+^, Cd^2+^	450	7.2	370.37, 625	[90]
Lignin-rich sulfated wood	–SO_3_H	-	Pb^2+^, Cu^2+^	1346.8, 476.625	3.5	331.52, 158.875	[91]

sPET—sulfonated poly (ethylene terephthalate); PET—poly (ethylene terephthalate).
